# Visual reading for [^18^F]Florzolotau ([^18^F]APN-1607) tau PET imaging in clinical assessment of Alzheimer’s disease

**DOI:** 10.3389/fnins.2023.1148054

**Published:** 2023-05-12

**Authors:** Huan-Chun Lin, Kun-Ju Lin, Kuo-Lun Huang, Shih-Hsin Chen, Tsung-Ying Ho, Chin-Chang Huang, Jung-Lung Hsu, Chiung-Chih Chang, Ing-Tsung Hsiao

**Affiliations:** ^1^Department of Nuclear Medicine, Chang Gung Memorial Hospital, Linkou Medical Center, Tao-Yuan, Taiwan; ^2^Department of Medical Imaging and Radiological Sciences, College of Medicine, Chang Gung University, Tao-Yuan, Taiwan; ^3^Healthy Aging Research Center, Chang Gung University, Tao-Yuan, Taiwan; ^4^Neuroscience Research Center, Chang Gung Memorial Hospital, Linkou Medical Center, Tao-Yuan, Taiwan; ^5^Department of Neurology, Linkou Chang Gung Memorial Hospital, Tao-Yuan, Taiwan; ^6^Department of Neurology, New Taipei Municipal TuCheng Hospital (Built and Operated by Chang Gung Medical Foundation), New Taipei City, Taiwan; ^7^Department of Neurology, Cognition and Aging Center, Institute for Translational Research in Biomedicine, Kaohsiung Chang Gung Memorial Hospital, Chang Gung University College of Medicine, Kaohsiung, Taiwan

**Keywords:** visual reading, [^18^F]Florzolotau, tau PET, Alzheimer’s disease, amyloid PET

## Abstract

**Introduction:**

Tau-targeted positron emission tomography (tau-PET) is a potential tool for the differential diagnosis of Alzheimer’s disease (AD) and to clarify the distribution of tau deposition. In addition to the quantitative analysis of tau-PET scans, visual reading supports the assessment of tau loading for clinical diagnosis. This study aimed to propose a method for visually interpreting tau-PET using the [^18^F] Florzolotau tracer and investigate the performance and utility of the visual reading.

**Materials and methods:**

A total number of 46 individuals with 12 cognitively unimpaired subjects (CU), 20 AD patients with mild cognitive impairment (AD-MCI), and 14 AD with dementia (AD-D) patients with both [^18^F]Florbetapir amyloid PET and [^18^F]Florzolotau tau PET scans were included. Clinical information, cognitive assessment, and amyloid PET scan results were recorded. For visual interpretation, a modified rainbow colormap was created and a regional tau uptake scoring system was proposed to evaluate the degree of tracer uptake and its spatial distribution within five cortical regions. Each region was scored on a scale of [0, 2] as compared to the background, and that resulted in a global scale range of [0, 10]. Four readers interpreted [^18^F]Florzolotau PET using the visual scale. The global and regional standardized uptake value ratios (SUVr) were also calculated for analysis.

**Results:**

The result indicates the average global visual scores were 0 ± 0 in the CU group, 3.43 ± 3.35 in the AD-MCI group, and 6.31 ± 2.97 in the AD-D group (*p* < 0.001). The consensus among the four observers on image scores was high with an intraclass correlation coefficient of 0.880 (95% CI: 0.767–0.936). The average global visual score was significantly associated with global SUVr (*r* = 0.884, *p* < 0.0001) and with the CDR-sum of box (*r* = 0.677, *p* < 0.0001).

**Conclusion:**

The visual reading method generated a visual score of [^18^F]Florzolotau tau-PET with good sensitivity and specificity to identify AD-D or CU individuals from the other patients. The preliminary result also showed that the global visual scores are significantly and reliably correlated with global cortical SUVr, and associated well with the clinical diagnosis and cognitive performance.

## Introduction

The accumulation of β-amyloid (Aβ) plaques and tau-containing neurofibrillary tangles is a hallmark of Alzheimer’s disease (AD) (Goedert and Jakes, 2005; Hardy, 2006). Previous studies (Arriagada et al., 1992; Braak and Braak, 1995; Cummings et al., 1996) have suggested that both the elevation of Aβ and neurofibrillary tangles are related to the severity of dementia in AD patients (Berg et al., 1998; Bennett et al., 2004; Nelson et al., 2009). Compared with cortical Aβ plaques, the phosphorylated tau aggregated in neurofibrillary tangles is more closely related to AD-related cognitive impairment and neurodegenerative changes (Duyckaerts et al., 1987; Dickson et al., 1997; Nelson et al., 2012). Therefore, accurate detection of the tau load of living patients is useful not only for establishing AD diagnosis and selecting the patient for targeted therapy (Fleisher et al., 2020) but also for predicting a patient’s potential for clinical progression. Recent advancements in selected tau tracers for PET imaging have facilitated the detection and quantification of tau pathology in living patients (Okamura et al., 2018).

In recent years, a number of tau tracers have been applied to the living human brain, including the first-generation tau tracers (Hashimoto et al., 2014; Harada et al., 2015; Betthauser et al., 2017; Hsiao et al., 2017; Stepanov et al., 2017) and the novel second-generation tau tracers, such as [^18^F]RO69558948, [^18^F]MK6240, and [^18^F]PI2620 (Walji et al., 2016; Declercq et al., 2017). Many studies have shown the tau burden measured from the tau PET imaging is highly correlated with neurodegeneration in AD (La Joie et al., 2020) and also exhibited high accuracy in differentiating AD from normal subjects and patients of other neurodegenerative disorders (Ossenkoppele et al., 2018; Mueller et al., 2020). Nevertheless, the first-generation tau tracers had some limitations, such as “off-target” binding; that is, the signal of the tau tracer comes from the binding of monoamine oxidase B (MAO-B) (Ng et al., 2017). Furthermore, most of these tracers have been found to exhibit strong binding affinity in the deep brain nucleus, which does not correspond to areas of high density of tangles in AD according to pathological studies (Braak and Braak, 1991). Therefore, there is an unmet need in dementia research for a tau-imaging agent with low off-target in the brain (Leuzy et al., 2019).

A first-generation tau tracer called [^11^C]PBB3 was developed in 2014, and shown to be effective in patients with AD and non-AD tauopathies for observing tau pathology in preclinical evaluations (Hashimoto et al., 2014; Ono et al., 2017). Recently, an ^18^F-labeled PBB3 derivative, [^18^F]Florzolotau (also known as [^18^F]APN1607 and [^18^F]PM-PBB3), was developed and shown to improve the imaging properties of [^11^C]PBB3 with broader accessibility and a higher signal-to-noise ratio for detecting tau pathologies in both human and animal studies (Hsu et al., 2020; Su et al., 2020; Lu et al., 2020; Tagai et al., 2021), and in non-AD tauopathies (Li et al., 2021; Liu et al., 2023).

However, the studies published recently have focused on the quantitative analysis of PET scans by tau tracers. While this method is suitable for research purposes, it can present challenges for clinical practice. Interpretation of visual scans may allow for a wider range of clinical applications, facilitate the use of tau PET by physicians, and thus may benefit patients. In the present study, we applied the [^18^F]Florzolotau tracer (1) to propose a reliable visual rating scale for the evaluation of [^18^F]Florzolotau PET (the visual scale), (2) to evaluate the reproducibility and how well the visual scale performs in differentiating AD with dementia (AD-D), Alzheimer’s disease with mild cognitive impairment (AD-MCI) and cognitively unimpaired patients (CU), (3) to study the accuracy of the visual scale compared to a quantitative method using SUVr (standard uptake value ratio).

## Materials and methods

### Ethical statement

The study protocol complied with the tenets of the Declaration of Helsinki and was approved by the Institutional Review Board of the Chang Gung Memorial Hospital (no: 201801834A0 and 201801833A0). All patients provided written informed consent to be included in the prospective study. All of the data were securely protected (by delinking identifying information from the main datasets), and access to the information was made available only to investigators and analyzed anonymously.

### Patients

We conducted the current study as a single-center investigation. The ages of all subjects were between 55 and 80 years. Alzheimer’s disease with mild cognitive impairment (AD-MCI) was defined as (1) amyloid-positive on the [^18^F]Florbetapir amyloid imaging, (2) clinical dementia rating (CDR) scale ≥0.5, and (3) independent daily life activities. Alzheimer’s disease with dementia (AD-D) was defined as (1) amyloid-positive on the [^18^F]Florbetapir amyloid imaging, (2) CDR scale ≥0.5, and (3) functionally impaired daily life activities. Cognitively unimpaired (CU) subjects were defined as (1) amyloid-negative on the ^18^F-Florbetapir amyloid imaging, (2) CDR scale = 0, and (3) independent daily life activities. Exclusion criteria for all subjects were history of other neurological disorders, history of stroke, severe progressive or unstable systemic diseases, recent cancer, and substance use disorder. Atypical AD patients were also excluded. Structural magnetic resonance imaging (MRI), amyloid PET, and tau PET scanning were performed in all subjects to fulfill the ATN diagnostic framework proposed in the NIA-AA 2018 research criteria (Albert et al., 2011; Jack et al., 2018). All individuals underwent cognitive evaluations, including the mini-mental state examination (MMSE), and the CDR scale. For disease severity, the CDR-sum of box (SOB) scores was employed. Categorization of subjects was based on both clinical presentations and cognitive test performance, and reached the consensus after discussion by neurologists, neuropsychologists, neuroradiologists, and experts in nuclear medicine. In brief, 12 Aβ (−) CU, 20 Aβ (+) AD-MCI, and 14 Aβ (+) AD-D subjects were included in this study.

### Image acquisition

Both [^18^F]Florbetapir and [^18^F]Florzolotau tracers were prepared and synthesized at the cyclotron facility of Chang Gung Memorial Hospital. All subjects underwent a 20 min [^18^F]Florzolotau scan at 90 min post-injection with an injection dose of 211.8 ± 25.1 MBq. All PET scans were performed in a Biograph mCT PET/CT system (Siemens Medical Solutions, Malvern, PA), and in a Discovery MI PET/CT system (GE Medical Systems, Milwaukee, MI). For the mCT PET/CT system, images were reconstructed using a 3-D OSEM algorithm of 4 iterations, 24 subsets, and post-smoothing using a Gaussian filter of 2 mm FWHM and zoom =3, and with CT-based attenuation correction. Scatter and random correction were also performed using the correction methods provided by the manufacturer. The reconstructed images had a matrix size of 400 × 400 × 109 and a voxel size of 1.02 × 1.02 × 2.03 mm^3^. For the Discovery MI PET/CT system, images were reconstructed using a VPHD algorithm of 4 iterations, 16 subsets with a matrix size 128 × 128 × 71 and a voxel size 2 × 2 × 2.79 mm^3^. The PET images were motion-corrected and averaged into 20 min static for later processing. Details on [^18^F]Florbetapir-PET acquisition and processing were described previously (Hsiao et al., 2013). Amyloid-PET positivity was evaluated by an experienced nuclear medicine specialist based on the visual assessment criteria for Florbetapir as described in the published guideline (Yang et al., 2012). The tau PET images were preprocessed with pre-optimized scanner-specific filters derived similarly as in Joshi et al.(2009) and from a previous phantom study so that all images from different scanners resulted in a unified resolution for further analysis.

For volumes of interest (VOI) delineation in the quantitation step, a T1-weighted magnetic resonance imaging (MRI) scan was acquired on a 3-T Siemens Magnetom TIM Trio scanner (Siemens Medical Solutions, Malvern, PA) for each subject.

### Imaging visual interpretation

#### Colormap adjustment

To consider the differential change of tau activity from normal, a colormap for visual reading modified from a rainbow colormap was created. The design of the color map consisted of three zones including background (deep blue), reference (green), and signal (red to yellow). To provide a guideline for setting up the colormap for the reference zone, the upper margin (or the center of the colormap) was initially set to mean + 2 standard deviations (SD) intensity of a normal group from a separate cohort (*n* = 42, from our previous studies), and this is approximately equal to 1.85 × *I*_crus_ (where *I*_crus_ indicates the mean intensity within the inferior cerebellar cortex for each subject). The color scale’s maximum was initially set to 2 times the upper margin of the reference zone (thus approximately equal to 3.7 × *I*_crus_ for each subject). Note that the above procedure in adjusting the colormap for each subject is simply applied as a starting point for a convenient colormap adjustment. For visual interpretation, the image was first loaded in transverse sections starting from the cerebellum to the vertex. Finally, the color scale’s maximum was adjusted to ensure the green color (reference zone) covered the whole cerebellum. From the above procedure, the result roughly displayed the signal in red to yellow color (signal zone) indicating regions with intensity levels approximately two standard deviations above the mean value of the cerebellar cortex derived from a separate normal cohort as described.

#### Regional tau uptake visual scoring

**Figure 1:**
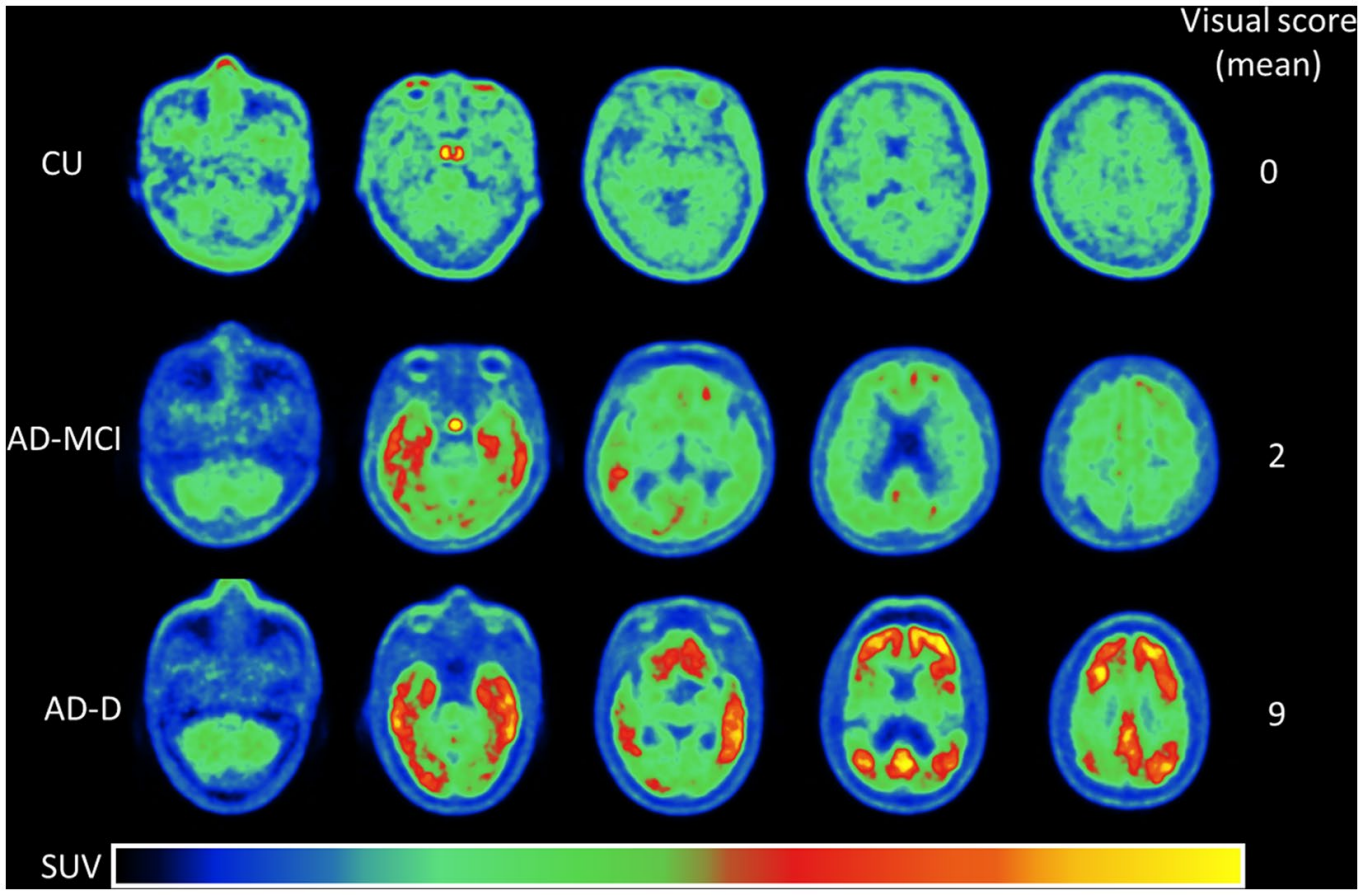
This shows the representative [^18^F]Florzolotau PET images from the cerebellum to the vertex including levels of the temporal, parietal, frontal, precuneus, and occipital lobes of cognitively unimpaired normal control (CU), Alzheimer’s disease with mild cognitive impairment (AD-MCI), and Alzheimer’s disease with dementia (AD-D) using a designed color scale. The global visual scores of the CU, AD-MCI, and AD-D were 0, 2, and 9.

The regional tau uptake scoring system in this work is modified from a previous study (Sonni et al., 2020) and developed based on the degree of tracer uptake in the regional and spatial distribution within the cortical area. The regions for visual reading including the temporal, parietal, frontal, precuneus, and occipital regions were selected based on our previous work (Hsu et al., 2020). Each region was scored on a 0 to 2 scale (0: no uptake, 1: mild uptake, 2: intense uptake) as compared to the background, and this resulted in a global scale range of 0–10. Note that a regional score of 0 is for the regional tau uptake (green color) lower than or equal to the background and 1 is for uptake only affecting a portion of the region or less than 50% area (total area on both sides is used as the denominator), while a score of 2 is for greater uptake affecting most of the region or more than 50% of the region. Specifically, the visual scoring system in this study considers the percentage of uptake from the regions of interest on both sides of the brain as a whole. As shown in Figure 1, examples of different regional and global scoring results were illustrated for representative tau images of CU, AD-MCI, and AD-D with different uptake levels on regional scales.

### Imaging quantitative analysis

All of the image data was processed and analyzed using the multimodality program PMOD (version 4.2, PMOD Technologies Ltd., Zurich, Switzerland). Images were spatially normalized into Montreal Neurological Institute space aided by individual MRI as described in previous work (Hsu et al., 2020). All VOIs modified from the automated anatomic labeling (AAL) atlas were applied for computing regional SUVr in comparison with the visual score including temporal, parietal, frontal, precuneus, occipital regions and the inferior cerebellar cortex (crus) reference region. After spatial normalization, we calculated SUVr images by referencing the inferior cerebellar cortex. There images were then used for further quantitative analysis. In addition to regional SUVr, a global SUVr was also calculated by taking the mean SUVr within the cortex area including temporal, frontal, parietal and occipital regions.

### Statistical analysis

Differences between groups were assessed with one-way ANOVA tests followed by Tukey *post hoc* tests for continuous variables, and Pearson’s chi-square test for categorical variables. Intraclass correlation coefficients (two-way random model, absolute agreement, single measure) were used to evaluate inter-reader agreement on the global visual scores. Pearson’s correlation coefficients were used to analyze the correlations between pairs of continuous variables. The statistical significance was set at *p* < 0.05 (two-tailed) for all analyses.

## Result

### Patient characteristics

The basic characteristics of the 46 individuals stratified by clinical diagnoses are presented in Table 1. We also conducted post-hoc pair-wise comparisons for each pair of groups on their characteristics and the associated global visual score, and the result was shown in the Supplementary Table S3. There were no significant differences in sex or years of education among the groups. The CU group was significantly younger (*p* = 0.004 compared to AD-MCI and *p* = 0.045 compared to AD-D), showed no evidence of amyloid deposition from amyloid PET images, and performed better on each neuropsychological examination (*p* < 0.001 for MMSE, CDR, and CDR-SOB) compared to both AD-MCI and AD-D groups. The AD-D group showed borderline or significant underperformance compared to the AD-MCI group on each neuropsychological examination (*p* = 0.066 for MMSE, *p* < 0.001 for CDR and CDR-SOB). No patients were diagnosed with other forms of dementia, such as frontotemporal dementia and dementia with Lewy bodies.

**Table 1:** General characteristics of study participants.

Characteristic	CU (*n* = 12)	AD-MCI (*n* = 20)	AD-D (*n* = 14)	ANOVA	ANCOVA^a^
				*p*-value	*p*-value
Age	64.6 ± 6.7	72.1 ± 4.5	71.1 ± 4.9	0.005	–
Female, *n* (%)	6 (50%)	13 (65%)	8 (57%)	0.699	0.697
Years of education	12.7 ± 4.7	9.4 ± 4.8	12.7 ± 4.1	0.065	0.156
Disease duration	0.0 ± 0.0	2.45 ± 1.73	3.86 ± 1.70	<0.001	<0.001
MMSE	28.7 ± 4.0	23.0 ± 3.2	20.7 ± 3.6	<0.001	<0.001
CDR	0.0 ± 0.0	0.5 ± 0.2	0.8 ± 0.3	<0.001	<0.001
CDR-sum of box	0.1 ± 0.4	2.3 ± 1.3	4.6 ± 1.6	<0.001	<0.001
Aβ-positive, *n* (%)	0 (0%)	20 (100%)	14 (100%)	<0.001	<0.001
Global visual score	0 ± 0	3.43 ± 3.35	6.31 ± 2.97	<0.001	<0.001

Unless otherwise indicated, data are presented in mean ± standard deviation. CU, cognitively unimpaired; AD-MCI, Alzheimer’s disease with mild cognitive impairment; AD-D, Alzheimer’s disease with dementia; ANOVA, analysis of variance; ANCOVA, analysis of covariance; MMSE, mini-mental state examination; CDR, clinical dementia rating; Aβ, beta-amyloid.

a Covariate adjusted for age.

### Visual assessment

A comparison of clinical subgroups stratified by clinical diagnosis showed that the average visual scores were 0 ± 0 in the CU group, 3.43 ± 3.35 in the AD-MCI group, and 6.31 ± 2.97 in the AD-D group (*p* < 0.001).

**Figure 2:**
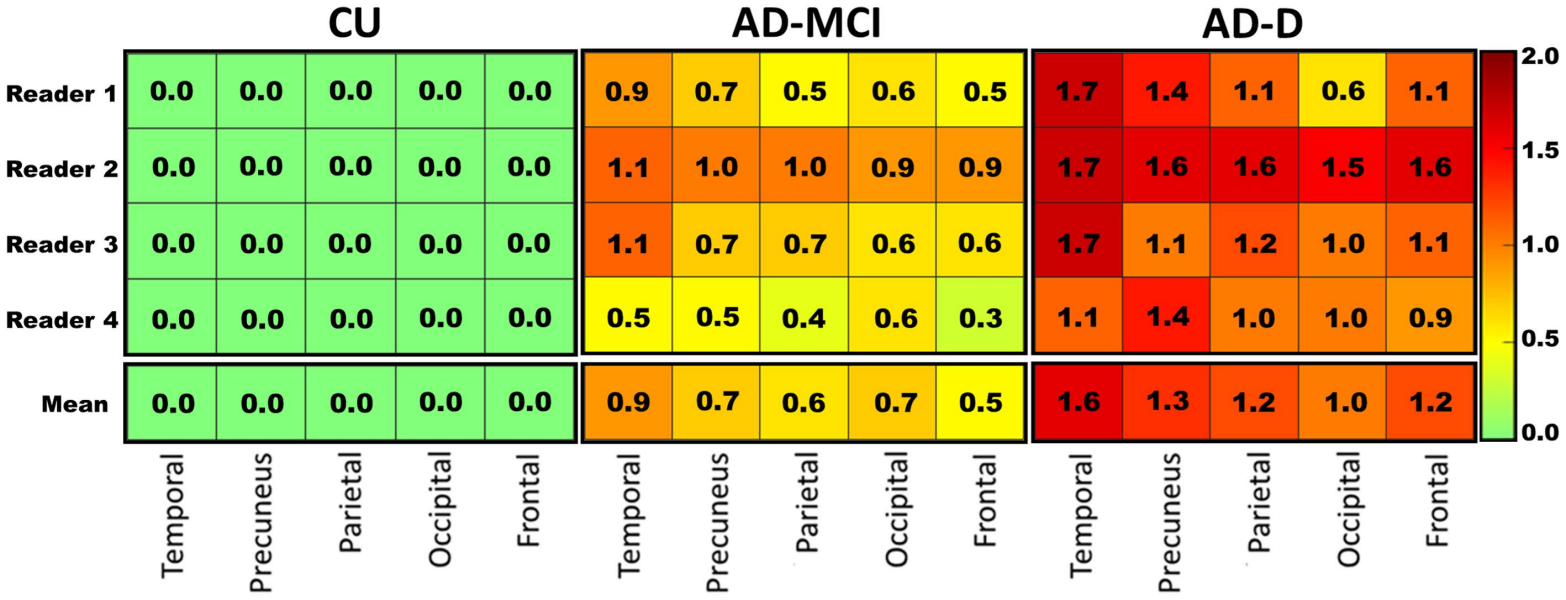
The heat map visualizing the average regional visual score rated by each reader (top 4 rows) in each clinical subgroup, and the mean score of the four readers (bottom row) in these regions. A clear pattern is visible where healthy controls in general tend to show lower scores while AD-D patients tend to show higher scores. Colors correspond to the score as shown in the right panel. CU: cognitively unimpaired subjects, *n* = 12; AD-MCI: Alzheimer’s disease with mild cognitive impairment, *n* = 20; AD-D: Alzheimer’s disease with dementia, *n* = 14.

Complementary analyses were run in five composite subregions in the brain. There were greater average visual scores in the temporal regions, followed by the precuneus and parietal lobes, and lower visual scores in the frontal and occipital regions. The temporal region read positive (>0) most often (60.9%), followed by the parietal (47.8%), while the frontal, precuneus, and occipital regions read positive less often (all 45.7%). Figure 2 shows the regional visual score distribution of patients classified into each subgroup, by different readers, and the average of all readers.

### Interobserver variation

The four readers had a high level of interobserver agreement (ICC = 0.880, 95%CI: 0.767–0.936) on the visual score, as determined by intraclass correlation coefficients [Shrout–Fleiss convention (2,1)]. The highest agreement was seen between reader 1 and reader 3 (ICC = 0.972) and the lowest was between reader 2 and reader 4 (ICC = 0.765, Table 2, Supplementary Table S1).

**Table 2:** Interobserver reliability.

Comparison of ROC curve in identifying cognitively unimpaired subjects from other patients between each reader				
A	B	AUC (A)	AUC (B)	*p*-value
Reader 1	Reader 2	0.9118	0.9191	0.8824
Reader 1	Reader 3	0.9118	0.9265	0.7454
Reader 1	Reader 4	0.9118	0.8113	0.1280
Reader 2	Reader 3	0.9191	0.9265	0.8787
Reader 2	Reader 4	0.9191	0.8113	0.1129
Reader 3	Reader 4	0.9265	0.8113	0.0758

Comparison of ROC curve in identifying AD-D patients from other subjects between each reader				
A	B	AUC (A)	AUC (B)	*p*-value
Reader 1	Reader 2	0.8158	0.8426	0.7661
Reader 1	Reader 3	0.8158	0.8181	0.9815
Reader 1	Reader 4	0.8158	0.8125	0.9728
Reader 2	Reader 3	0.8426	0.8181	0.7856
Reader 2	Reader 4	0.8426	0.8125	0.7439
Reader 3	Reader 4	0.8181	0.8125	0.9548

ROC, receiver operating characteristic; AUC, area under curve; AD-D, Alzheimer’s disease with dementia.

### Visual score vs. quantitation

**Figure 3:**
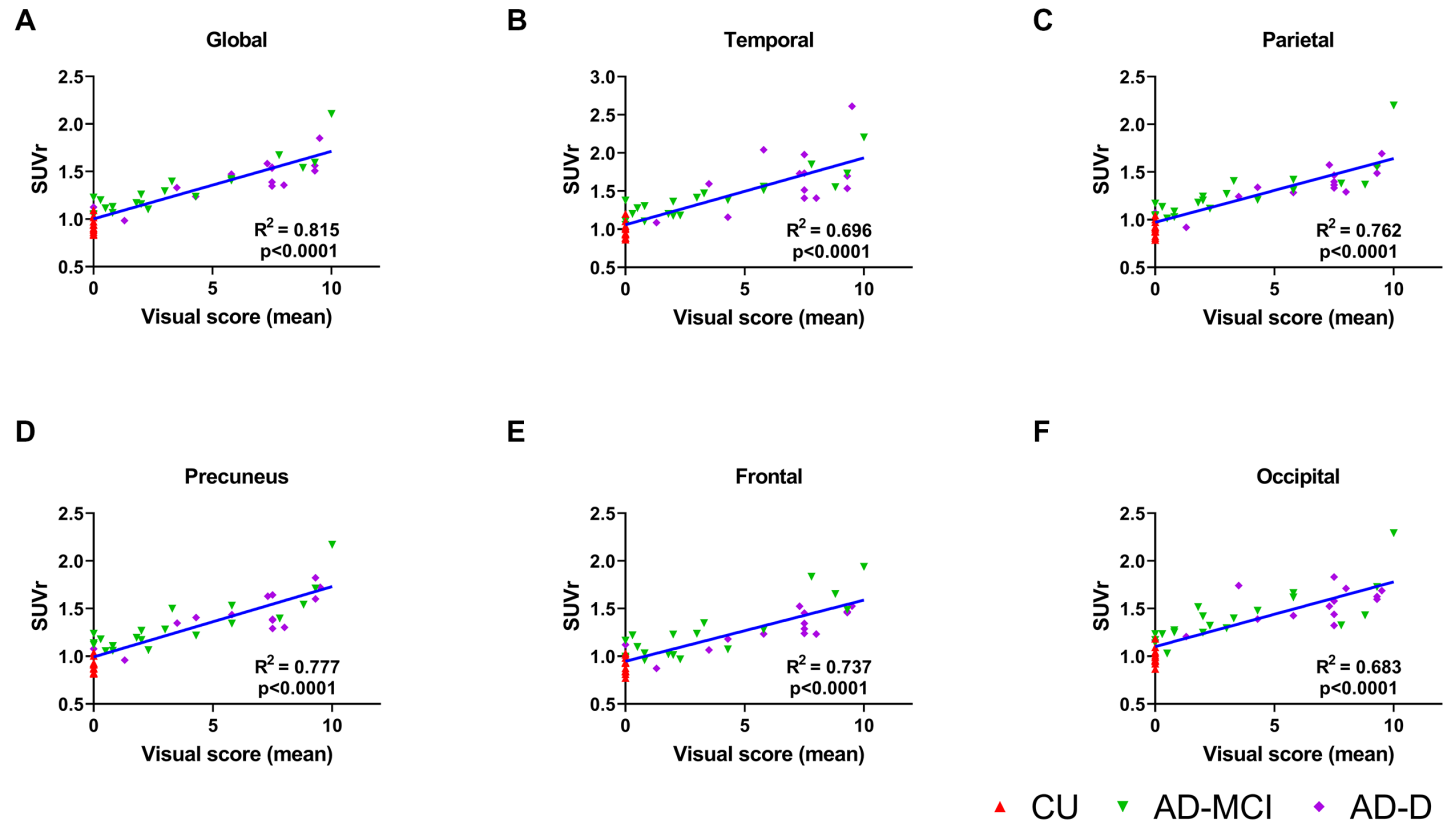
**(A)** This plot displays correlations between the mean global visual score of 4 readers and global cortical standardized uptake value ratio (SUVr) for the groups of CU (red), AD-MCI (green) and AD-D (purple). The correlations of the mean regional visual score of 4 readers and regional cortical standardized uptake value ratio (SUVr) are shown in **(B)**–**(F)** for temporal, parietal, precuneus, frontal and occipital lobes, separately.

The average global visual score was significantly associated with global [^18^F]Florzolotau tau-PET SUVr (Pearson’s correlation coefficient, *R*^2^ = 0.815, *p* < 0.0001, Figure 3A). The association between the global tau-PET SUVr and the individual global visual score of each reader (Supplementary Figure S1) was also explored, showing that the correlations were also high (highest *R*^2^ = 0.8, lowest *R*^2^ = 0.733; all *p* < 0.0001).

When comparing quantitative tau-PET SUVr values in different regions of the brain, the highest SUVr values were in the temporal lobes and the lowest SUVr values were in the frontal lobe, which is consistent with the result of the visual score. Furthermore, the correlations between the average visual scores and the tau-PET SUVr in these five regions were investigated, and the results show a high correlation in all regions (frontal *R*^2^ = 0.737, precuneus *R*^2^ = 0.777, temporal *R*^2^ = 0.696, parietal *R*^2^ = 0.762, occipital *R*^2^ = 0.683; all *p* < 0.0001, Figures 3B–F). In addition, each reader’s visual score also displayed a moderate correlation with the calculated global SUVr as shown in the Supplementary Figure S1.

### Visual score accuracy

**Figure 4:**
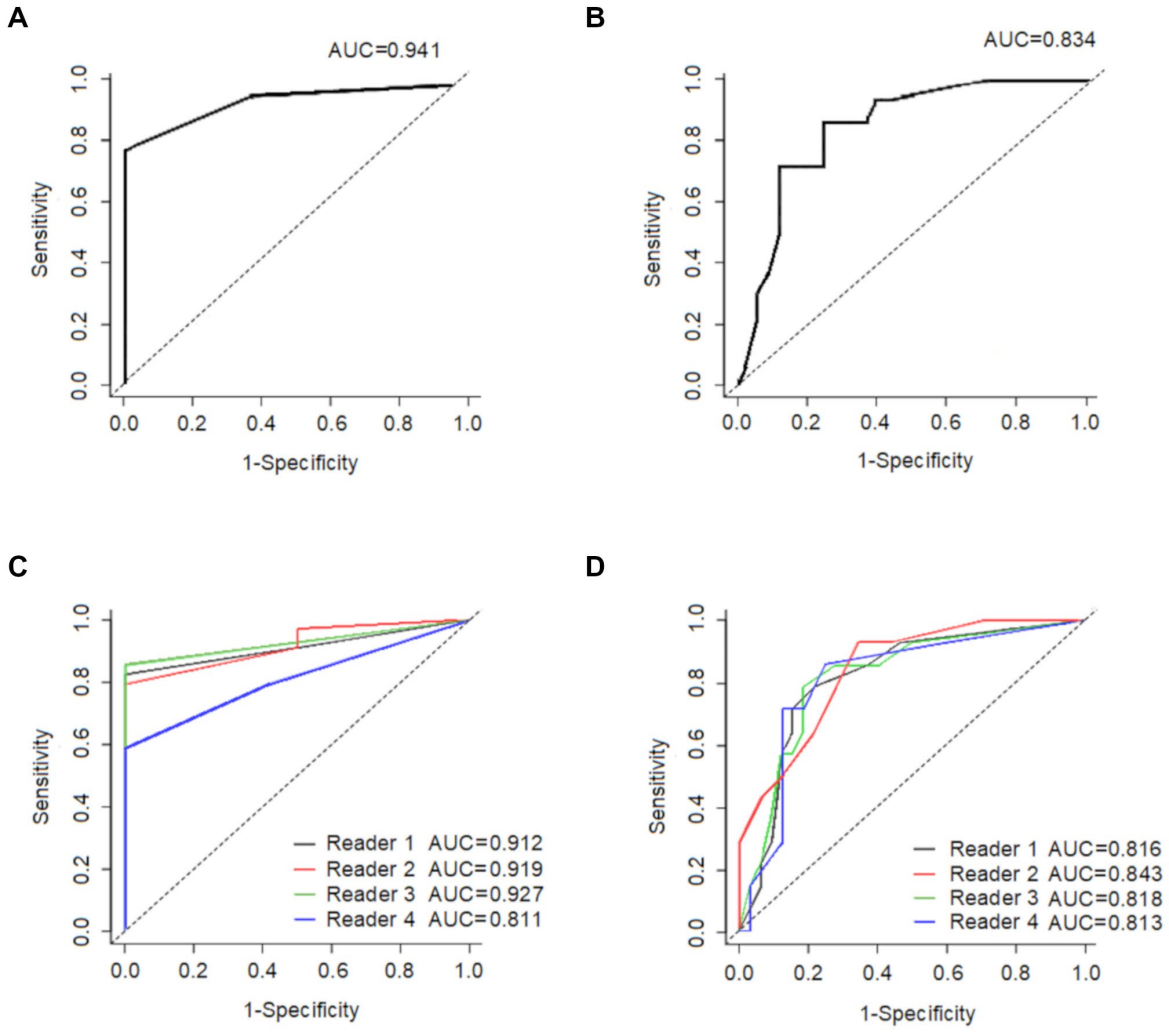
This shows the ability of the average global visual score to **(A)** distinguish CU (*n* = 12) from all other participants (*n* = 34), and **(B)** distinguish AD-D (*n* = 14) from all other participants (*n* = 32), The ability of each reader’s global visual score is displayed in **(C)** to distinguish CU (*n* = 12) from all other participants (*n* = 34), and in **(D)** to distinguish AD-D (*n* = 14) from all other participants (*n* = 32). CU: cognitively unimpaired subjects; AD-D: Alzheimer’s disease with dementia.

All CU group subjects (100%) scored “0” compared to a minority of the AD-MCI (15%) and AD-D (8%) patients. Receiver operating characteristic (ROC) curve analyses (Figure 4) were used to determine if the visual score could: (1) distinguish the AD-D group from the other patients and (2) distinguish the CU group from the other patients.

An optimal cut-off score of 1.0 had a sensitivity of 82.4% and a specificity of 100% in differentiating CU patients from the other patients with the AD-spectrum, and the resulted area under the ROC curve (AUC) was 0.94. In comparison, the cut-off visual score of 4.0 had a sensitivity of 85.7% and a specificity of 78.1% in distinguishing AD-D patients from other patients within the AD-spectrum, resulting in an AUC of 0.83.

### Visual score vs. clinical performance and other characteristics

There were moderate correlations between the visual scores and CDR (*R*^2^ = 0.271, *p* < 0.001), and CDR-sum of box (*R*^2^ = 0.458, *p* < 0.001) but mild correlation between visual scores and MMSE (*R*^2^ = 0.199, *p* = 0.002). There was no correlation between visual scores and other subject characteristics (Supplementary Table S2).

**Figure 5:**
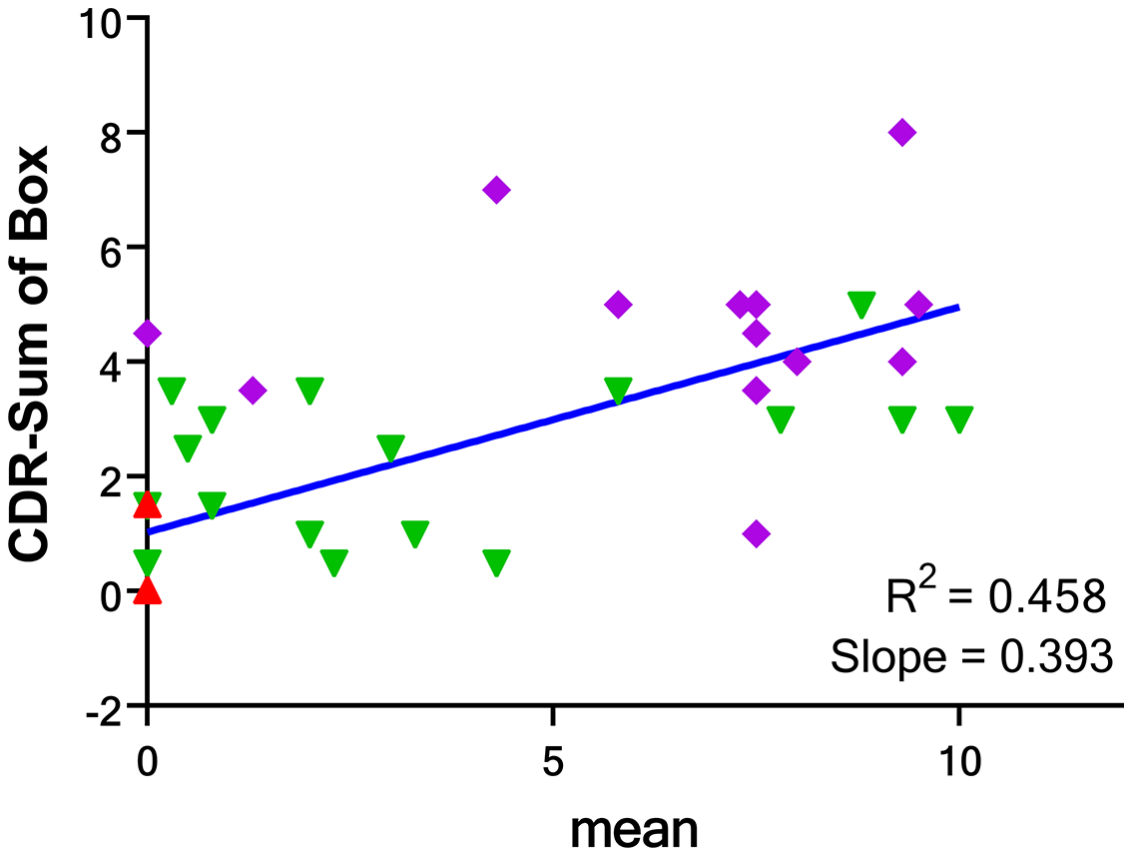
Correlations between the mean global visual score of 4 readers and CDR-SOB for the groups of CU (red), AD-MCI (green) and AD-D (purple). CDR-SOB: clinical dementia rating—sum of box. CU: cognitively unimpaired subjects; AD-MCI: Alzheimer’s disease with mild cognitive impairment; AD-D: Alzheimer’s disease with dementia.

The correlation between the mean visual score from the four readers and the CDR-SOB is shown in Figure 5, while the correlation between the individual visual score of the four readers and the CDR-SOB is displayed in the Supplementary Figure S2. The visual scores of each reader showed high to moderate correlations with the CDR-SOB (highest *R*^2^ = 0.465, lowest *R*^2^ = 0.376; all *p* < 0.0001).

## Discussion

Although amyloid and tau pathologies may start separately (Mungas et al., 2014; Jack et al., 2018), they are closely related to each other during the symptomatic stages of AD (Monsell et al., 2013). In this research, we proposed a visual scoring scale from clinically obtained tau-PET scans for detecting the degree of tau accumulation in the brain.

The main result of this single-center study is that the visual inspection of [^18^F]Florzolotau tau uptake distribution can provide accurate diagnosis for differentiating AD pathology from cognitively unimpaired subjects. The results show that our visual technique for interpreting [^18^F]Florzolotau tau-PET is highly correlated with SUVr measurement and is related to clinical diagnosis. The mean visual scores were significantly different between the AD-D, AD-MCI and CU groups, indicating that the visual scoring scale can be sued to distinguish between these diagnostic groups.

We evaluated the performance of the visual scale against the quantitative SUVr method because the SUVr has been validated in various studies (Clark et al., 2012; O’brien et al., 2014; Joshi et al., 2015; Harn et al., 2017). Following the previous studies, we found that the quantitative SUVr of AD-D patients was significantly higher than those of AD-MCI and the CU groups. When comparing the visual scale with the quantitative SUVr method, the visual scale demonstrated comparable diagnostic precision for identifying AD-D patients or cognitively unimpaired subjects from other patients. Specifically, a cut-off visual score of 4 could significantly differentiate the AD-D patients from the other patients, and a cut-off score of 1 could distinguish the cognitively unimpaired subjects from the other (AD-MCI and AD-D) patients. The visual scale we proposed (global score) allowed the differentiation of cognitively unimpaired subjects from other patients with a sensitivity of 82.4% and a specificity of 100%; and differentiated AD-D from other participants with 85.7% sensitivity and 78.1% specificity. The findings of our study suggest that [^18^F]Florzolotau tau-PET visual reads are feasible and can identify tau pathology with high accuracy, supporting its translation to clinical settings as an aid in the assessment of tau accumulation in the brain. These preliminary findings suggest that visual reading could detect tau aggregation in different brain regions, supporting the value of our visual scoring tool in capturing the extent of tau burden.

The AUC of our visual scores for distinguishing AD-D from other patients was 0.834, compared to the AUC of 0.949 reported by a recent [^18^F]Florzolotau ([^18^F]APN1607) study using the SUVr method (Zhang et al., 2021). However, it is important to note that the purpose of that study was to separate AD with dementia from the health controls, not to distinguish AD with dementia from other patients including AD-MCI.

While each reader’s experience and potential bias can unavoidably impact visual interpretation, we found an acceptable inter-reader agreement (ICC ≥ 0.77), suggesting that the visual scale may be reliable and useful in a clinical setting.

In our previous AD study (Hsu et al., 2020), the clinical performance and quantification of [^18^F]Florzolotau tau accumulation showed a close relationship. In our current results, the global visual scores in [^18^F]Florzolotau tau-PET images from AD-associated regions showed significant positive correlations with the CDR-SOB scores (*p* < 0.0001), demonstrating that the increasing tau burden identified by the visual reading score closely correlated with decreasing cognition and increasing disease severity.

Notably, from a clinical point of view, a visual scale has several advantages compared to a quantitative method. The quantitative method can be time-consuming, and the actual value can vary depending on many factors, such as the ROI configuration, tau-PET and CT data processing, and the essential software. Moreover, the quantitative method may require specialized expertise and equipment, which might only be available in a medical center, limiting its accessibility and practicality in routine clinical settings. In contrast, a visual rating scale can be implemented as a fast-diagnostic tool adjunct to the clinical tau-PET scan evaluation without additional complex processing.

This study shows strong correlations between mean visual score and global and regional SUVr values. For comparison, the correlation analysis between global/regional SUVr values and CDR-sum of box were illustrated in the Supplementary Table S7 with good correlations of global and regional SUVr values with CDR-SOB. As compared with the mean visual score result as in Figures 3, a slightly higher correlation with CDR-SOB than the SUVr result (*R*^2^ = 0.458 for the global visual score and *R*^2^ = 0.348 for the global SUVr, respectively). In addition, the ROC analyses for the global SUVr were also performed and the results were shown in the Supplementary Figure S6 with the AUC of 0.985 for differentiating CU from other patients and 0.783 for AD-D from other subjects. As compared with the result for the mean visual score in Figure 5, SUVr performs slightly better than the proposed visual scoring method in classifying CU from other subjects (AUC = 0.985 for SUVr and AUC = 0.941 for visual score), while the visual score result performs slightly better in classifying AD-D from others (AUC = 0.783 for SUVr and AUC = 0.834 for visual score). From the above results and a small number of cases, although the proposed visual reading method seems to generate compatible results with the semiquantitative SUVr, further research with a larger sample size is needed to determine whether this method conveys prognostic information. Moreover, longitudinal data should be collected to evaluate the prognostic value of the visual scale for the tau tracer of [^18^F]Florzolotau compared to quantitative methods.

The applications of tau PET in differentiating AD-D from other neurodegenerative cognitive disorders such as frontotemporal dementia (FTD) and dementia with Lewy bodies (DLB) have been explored before (Gomperts et al., 2016; Ossenkoppele et al., 2018; Kroth et al., 2019; Tsai et al., 2019; Mak et al., 2021). Investigations of tau PET in FTD patients using [^18^F]Florzolotau (or [^18^F]PM-PBB3) have been reported as well (Su et al., 2020; Tagai et al., 2021). Thus, due to the capability of detecting both 3R and 4R tauopathies, the tau PET [18F]Florzolotau may play a role in the differential diagnosis of other neurodegenerative cognitive disorders such as FTD and DLB. This article is a preliminary study of a visual scoring system to evaluate its reliability and association with disease severity. To expand on these findings, we plan to recruit non-AD cases to determine if this scoring system can provide more information and help classify Alzheimer’s disease continuum and other dementia patients.

Our analysis also revealed higher visual scores in certain sub-regions, such as the lateral temporal regions, indicating that these areas may be more vulnerable to tau accumulation associated with amyloid. The Braak hypothesis was generally supported by the observed patterns of visual scores in the AD-MCI group. As the disease progressed in the AD-D group, the burden of tau was elevated in all regions. However, we did notice the existence of different uptake pattern from the typical temporoparietal distribution among the AD-MCI and AD-D groups. Please see the heatmap in the Supplementary Figure S4 for the heatmap result where some subjects are with atypical uptake patterns. Moreover, according to the pathological development of Alzheimer’s disease, there may be some areas that are affected by tau earlier in the course of the disease before clinical symptoms appear. With further research, elevated visual scores of tau deposition in these regions may help identify patients at high risk of Alzheimer’s disease.

The PET images were scanned from two different scanners, and to reduce the scanner effect, we have performed the data harmonization processing as in (Joshi et al., 2009) to achieve a unified resolution for images from the two scanners. The main goal of this study is to propose a visual reading method for [18F]Florzolotau PET imaging. A thorough study of the scanner effect needs to be conducted using the same subjects scanned from different scanners or using a large sample size. Nevertheless, as indicated in the Supplementary Tables S4–S6, the number of subjects scanned with those two different scanners per group was not evenly distributed, and there are more cases in the AD-MCI group scanned from the Discovery MI PET/CT system, while slightly more cases in the AD-D group using the Biograph mCT PET/CT system. However, the patient characteristics of the two scanners did not show significant differences in each subject group (Supplementary Tables S4–S6). Similarly, there were no significant differences in visual score and SUVr between two scanners in the same subject group. In particular, for the CU groups scanned from these two scanners, compatible values of the mean visual scores and global and regional SUVr’s were observed.

Some limitations in the current study must also be noted. First, the sample size was relatively small, which can minimize the statistical power of the visual reading score for the classification of AD-D/AD-MCI/CU patients. The second limitation of this study is that the final diagnosis was only confirmed by clinical evaluations without pathological confirmation of amyloid or tau positivity for AD diagnosis. Third, although the age selection criteria were set the same for all groups in our study, the CU subjects were still younger than the AD-D and AD-MCI patients. We have also included age as the covariate to compare the cognitive performance among the three subject groups in Table 1, and the difference in cognitive results remained significant. As age is one of the major risk factors for Alzheimer’s disease, the visual reading scoring method for [^18^F]Florzolotau proposed in this study has to be validated in a larger sample size with wider age range before general application. Further, due to the lack of follow-up for observing changes in dementia status over time, it is impossible to know whether the visual scoring system is also related to clinical deterioration over time.

However, our study’s strengths include using better color scales to visually distinguish areas with the accumulation of tau. The color scale we used can quickly switch between the two colors, so readers can easily and immediately see which areas have high tau protein accumulation. Our tau-PET visual interpretation method was developed using improved color scales and incorporates a visual rating composite score, which has shown superior performance in classifying AD spectrum patients compared to traditional visual scoring methods.

## Conclusion

In conclusion, our study shows that the proposed visual scale of [^18^F]Florzolotau ([^18^F]APN1607) tau-PET is strongly associated with SUVr quantification and clinical diagnosis, suggesting that the visual scale of tau-PET is a robust and clinical application to distinguish AD with dementia patients from other older adults with cognitive decline. Visual interpretation of tau-PET imaging can reflect quantitative tau measurements evaluated by PET, thus representing another promising alternative to the quantitative PET method in clinical settings. The inter-reader reliability of our visual scoring is strong, indicating that the proposed visual method is reproducible and therefore potentially for clinical application to evaluate tau concentrations. Future studies should evaluate the potential and limitations of tau-PET and the proposed visual reading approach by using longitudinal PET and cognitive data, and the prognostic role of this method should be further clarified in clinical settings.

## Data availability statement

The raw data supporting the conclusions of this article will be made available by the authors, without undue reservation.

## Ethics statement

The studies involving human participants were reviewed and approved by Institutional Review Board of the Chang Gung Memorial Hospital. The patients/participants provided their written informed consent to participate in this study.

## Author contributions

K-JL and I-TH: conception, design of the work, and revision of the manuscript. H-CL, K-JL, and I-TH: analysis of data and writing of the manuscript. H-CL, K-JL, S-HC, and T-YH: interpretation of images. K-LH, C-CH, J-LH, and C-CC: data collection. K-JL: funding acquisition. All authors contributed to the article and approved the submitted version.

## Funding

This study was carried out with financial support from the National Science and Technology Council, Taiwan (MOST-109-2314-B-182A-043-MY3, MOST-109-2314-B-182-019-MY3, NSTC-111-2623-E-182-001-NU, and NSCT-112-2263-E-182A-001-NU), grant from the Ministry of Health and Welfare of Taiwan (MOHW111-TDU-B-212-134005), and grants from the Research Fund of Chang Gung Memorial Hospital (CMRPG3J0374, CMRPG3J0364, BMRP-488, CMRPG3J1571 and CMRPD1M0111-2).

## Conflict of interest

The authors declare that the research was conducted in the absence of any commercial or financial relationships that could be construed as a potential conflict of interest.

## Publisher’s note

All claims expressed in this article are solely those of the authors and do not necessarily represent those of their affiliated organizations, or those of the publisher, the editors and the reviewers. Any product that may be evaluated in this article, or claim that may be made by its manufacturer, is not guaranteed or endorsed by the publisher.
