# Supplementary material for: Visual reading for [18F]Florzolotau ([18F]APN-1607) tau PET imaging in clinical assessment of Alzheimer’s disease
Source: Front Neurosci. 2023 May 12;17:1148054. doi: 10.3389/fnins.2023.1148054 (PMC10213356; doi:10.3389/fnins.2023.1148054)
Supplement: Supplementary file 1 [file Data_Sheet_1.PDF]

Visual reading for [^{18}F]Florzolotau ([^{18}F]APN-1607) tau PET imaging in clinical assessment of Alzheimer's Disease

Huan-Chun Lin[1 ǂ], Kun-Ju Lin[1,2,3,4 ǂ], Kuo-Lun Huang[5], Shih-Hsin Chen[1], Tsung-Ying Ho[1], Chin-Chang Huang[5], Jung-Lung Hsu[5], Chiung-Chih Chang[6], Ing-Tsung Hsiao[1,2,3,4 *],

Supplementary materials:

sTable 1
This shows the interobserver reliability from the intraclass correlation coefficients between each reader.

Comparison	Intraclass correlation coefficients	95% CI	Significance
Observer 1/2	0.842	0.509-0.934	<0.001
Observer 1/3	0.972	0.946-0.985	<0.001
Observer 1/4	0.947	0.892-0.973	<0.001
Observer 2/3	0.883	0.663-0.949	<0.001
Observer 2/4	0.765	0.290-0.903	<0.001
Observer 3/4	0.918	0.792-0.962	<0.001

Abbreviation: CI, confidence interval

sTable 2
Correlation between visual score and other characteristics

Characteristics	R squared	p value
Age	0.002	0.792
Female, n (%)	0.001	0.839
Years of education	0.001	0.821
Disease duration	0.011	0.562
MMSE	0.199	0.002
CDR	0.271	<0.001
CDR sum of box	0.458	<0.001

Abbreviation: MMSE, mini-mental state examination; CDR, Clinical Dementia Rating

sTable3

Post-hoc analysis for subject characteristics.

	Post-hoc analysis				
				95% confidence interval	
Characteristic	A	B	P value	Lower bound	Upper bound
Age	CU	AD-MCI	0.004	-11.23	-1.87
	AD-MCI	AD-D	0.836	-3.41	5.51
	CU	AD-D	0.030	-10.54	-0.46
Disease duration	CU	AD-MCI	<0.001	-3.46	-1.44
	AD-MCI	AD-D	0.074	-2.92	0.10
	CU	AD-D	<0.001	-5.09	-2.62
MMSE	CU	AD-MCI	<0.001	2.88	8.45
	AD-MCI	AD-D	0.105	-0.38	4.95
	CU	AD-D	<0.001	5.01	10.89
CDR	CU	AD-MCI	<0.001	-0.61	-0.31
	AD-MCI	AD-D	0.005	-0.49	-0.08
	CU	AD-D	<0.001	-0.95	-0.54
CDR-sum-of-box	CU	AD-MCI	<0.001	-3.06	-1.28
	AD-MCI	AD-D	0.001	-3.78	-0.86
	CU	AD-D	<0.001	-5.80	-3.19
Global visual score	CU	AD-MCI	0.001	-5.38	-1.48
	AD-MCI	AD-D	0.038	-5.63	-0.12
	CU	AD-D	<0.001	-8.46	-4.15

Abbreviation: CU, cognitively unimpaired; AD-MCI, Alzheimer's disease with mild cognitive impairment; AD-D, Alzheimer's disease with dementia; MMSE, mini-mental state examination; CDR, Clinical Dementia Rating

sTable 4. Differences of characteristics, visual score and SUVr between scanners in CU group

	Scanner		
	GE MI (n=7)	Siemens mCT (n=5)	P value
Age	67.6 ± 6.4	62.6 ± 6.6	0.218
Female, n (%)	2 (29%)	4(80%)	0.079
Years of education	11.4 ± 4.9	14.4 ± 4.3	0.304
Disease duration	0 ± 0	0 ± 0	not available
MMSE	28.1 ± 2.5	29.4 ± 0.9	0.32
CDR	0.1 ± 0.2	0 ± 0	0.424

CDR-sum of box		0.2 ± 0.5	0 ± 0	0.424
Visual score	Global	0 ± 0	0 ± 0	not available
	Frontal	0 ± 0	0 ± 0	not available
	Parietal	0 ± 0	0 ± 0	not available
	Precuneus	0 ± 0	0 ± 0	not available
	Temporal	0 ± 0	0 ± 0	not available
	Occipital	0 ± 0	0 ± 0	not available
SUVr	Global	0.94 ± 0.09	0.94 ± 0.07	0.876
	Frontal	0.90 ± 0.09	0.91 ± 0.07	0.755
	Parietal	0.90 ± 0.08	0.90 ± 0.08	0.994
	Precuneus	0.91 ± 0.09	0.91 ± 0.08	0.999
	Temporal	0.99 ± 0.11	0.97 ± 0.07	0.751
	Occipital	1.00 ± 0.11	1.00 ± 0.07	0.910

Unless otherwise indicated, data are presented in mean ± standard deviation

Abbreviation: CU, cognitively unimpaired; MMSE, mini-mental state examination; CDR, Clinical Dementia Rating

sTable 5. Differences of characteristics, visual score and SUVr between scanners in AD-MCI group

		Scanner		
		GE MI (n=17)	Siemens mCT (n=3)	P value
Age		72.3 ± 4.6	70.7 ± 5.0	0.581
Female, n (%)		11 (65%)	2 (67%)	0.948
Years of education		8.9 ± 4.5	12.3 ± 6.5	0.266
Disease duration		2.5 ± 1.9	2.3 ± 0.6	0.903
MMSE		22.9 ± 3.0	23.3 ± 4.7	0.850
CDR		0.5 ± 0.2	0.5 ± 0.0	1.000
CDR-sum of box		2.3 ± 1.3	2.3 ± 1.6	0.957
Visual score	Global	3.3 ± 3.4	4.1 ± 3.7	0.704
	Frontal	0.5 ± 0.7	0.8 ± 1.0	0.592
	Parietal	0.6 ± 0.7	0.9 ± 0.9	0.496
	Precuneus	0.7 ± 0.8	1.0 ± 1.0	0.534
	Temporal	0.8 ± 0.7	1.1 ± 1.0	0.594
	Occipital	0.7 ± 0.8	0.3 ± 0.6	0.446
SUVr	Global	1.30 ± 0.27	1.37 ± 0.26	0.479
	Frontal	1.22 ± 0.27	1.38 ± 0.40	0.546

	Parietal	1.27 ± 0.28	1.24 ± 0.13	0.842
	Precuneus	1.32 ± 0.29	1.26 ± 0.12	0.921
	Temporal	1.39 ± 0.28	1.48 ± 0.33	0.546
	Occipital	1.41 ± 0.30	1.34 ± 0.12	0.704

Unless otherwise indicated, data are presented in mean ± standard deviation

Abbreviation: AD-MCI, Alzheimer's disease with mild cognitive impairment; MMSE, mini-mental state examination; CDR, Clinical Dementia Rating

sTable 6. Differences of characteristics, visual score and SUVr between scanners in AD-D group

		Scanner		
		GE MI (n=5)	Siemens mCT (n=9)	P value
Age		69.0 ± 6.4	72.1 ± 3.9	0.276
Female, n (%)		2 (40%)	6 (67%)	0.334
Years of education		15.4 ± 2.2	11.2 ± 4.2	0.062
Disease duration		4.2 ± 1.8	3.7 ± 1.7	0.595
MMSE		21.0 ± 4.0	20.6 ± 3.6	0.834
CDR		0.8 ± 0.3	0.8 ± 0.3	0.884
CDR-sum of box		4.9 ± 1.3	4.4 ± 1.9	0.651
Visual score	Global	5.4 ± 3.3	6.8 ± 2.8	0.395
	Frontal	1.0 ± 0.7	1.3 ± 0.9	0.581
	Parietal	1.1 ± 0.8	1.3 ± 0.8	0.819
	Precuneus	1.3 ± 0.9	1.4 ± 0.8	0.847
	Temporal	1.3 ± 0.9	1.7 ± 0.4	0.257
	Occipital	0.7 ± 0.7	1.2 ± 0.7	0.158
SUVr	Global	1.37 ± 0.18	1.44 ± 0.24	0.606
	Frontal	1.27 ± 0.13	1.30 ± 0.22	0.799
	Parietal	1.33 ± 0.15	1.38 ± 0.23	0.898
	Precuneus	1.36 ± 0.20	1.47 ± 0.26	0.519
	Temporal	1.51 ± 0.36	1.68 ± 0.44	0.699
	Occipital	1.46 ± 0.26	1.55 ± 0.17	0.364

Unless otherwise indicated, data are presented in mean ± standard deviation

Abbreviation: AD-D, Alzheimer's disease with dementia; MMSE, mini-mental state examination; CDR, Clinical Dementia Rating

sTable 7. Correlations of global and regional SUVr with CDR-SOB

		R^2	P-value
SUVr vs CDR-SOB	Global	0.348	<0.0001
	Frontal	0.363	<0.0001
	Parietal	0.337	<0.0001
	Precuneus	0.311	<0.0001
	Temporal	0.292	0.0001
	Occipital	0.297	<0.0001

CDR-SOB: Clinical Dementia Rating – Sum of Box

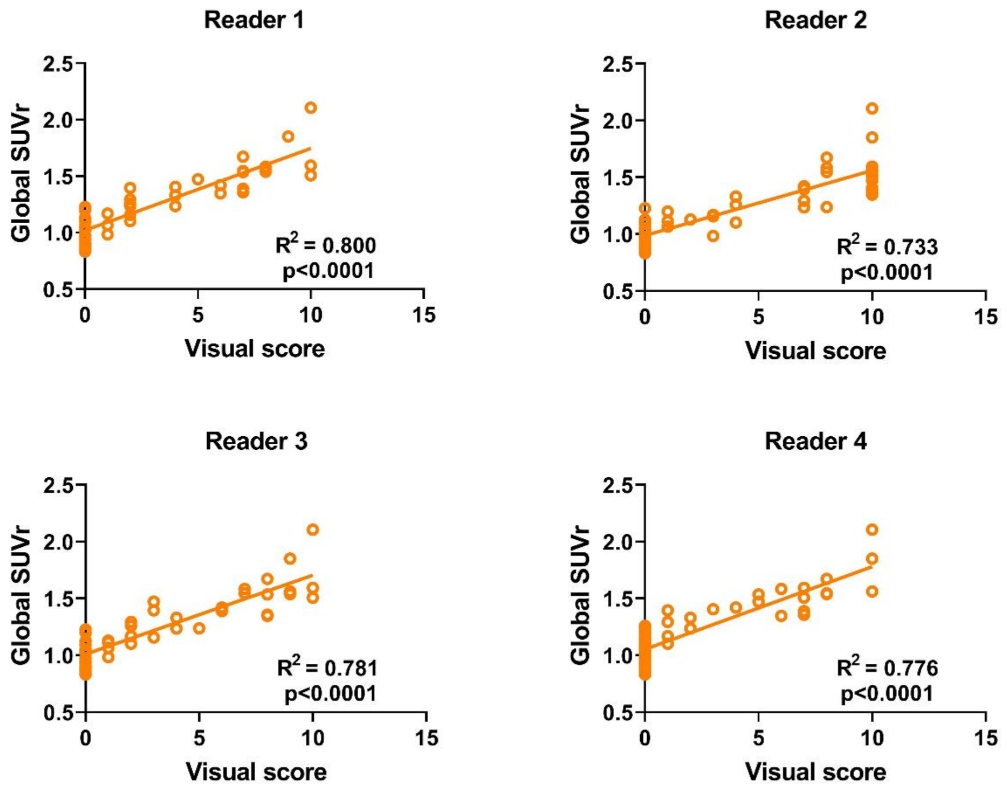

sFigure 1: This shows the correlations between each reader's global visual score and global cortical standardized uptake value ratio (SUVr). The result displays high correlation between the visual score and the quantitation results for each reader (highest R^2=0.8, lowest R^2= 0.733; all p<0.0001).

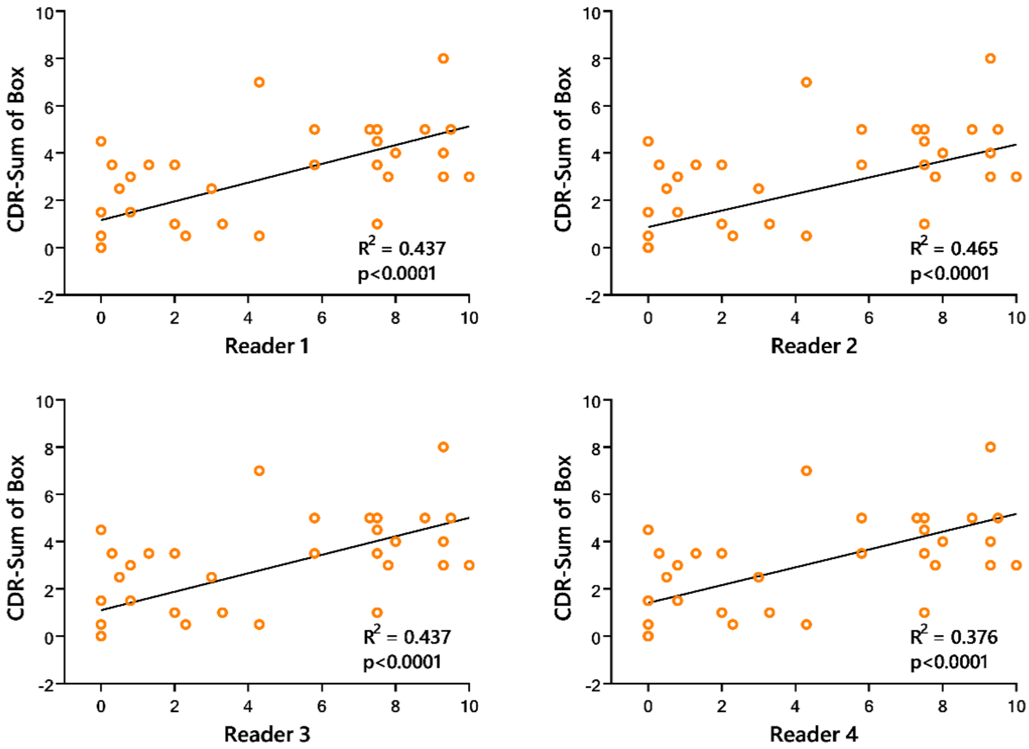

sFigure 2: This shows the correlations between each reader's global visual score obtained from the visual reading method and the clinical performance measured by the CDR-SOB (sum-of-box). The result indicates relatively high correction between the visual reading scores and the CDR-SOB for each reader. CDR: clinical dementia rating.

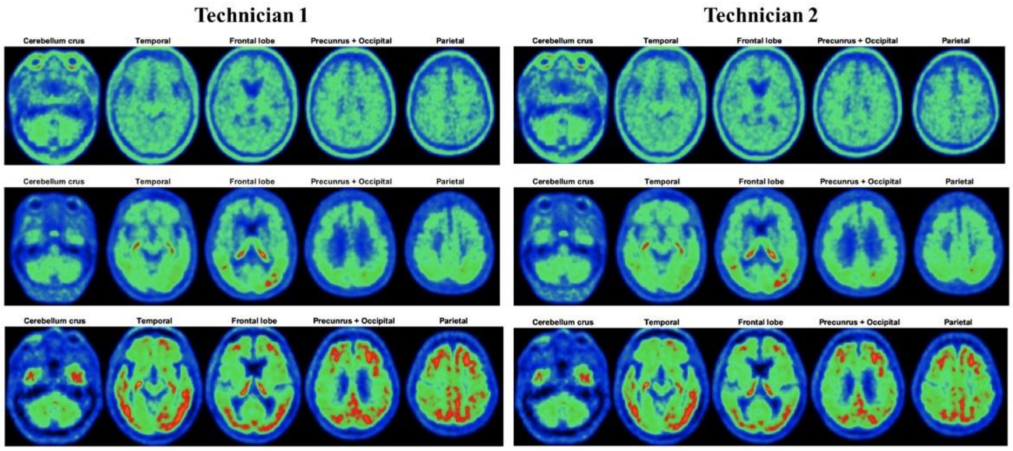

sFigure 3. This figure displays the adjusted 18F-Florzolotau PET images of three subjects from two technicians based on the colormap adjustment as described in the manuscript. The same PET images as adjusted by both technicians are shown in the same row, and the results look similar from both technicians. Further study is needed to investigate the variation from colormap adjustment in this proposed visual reading method.

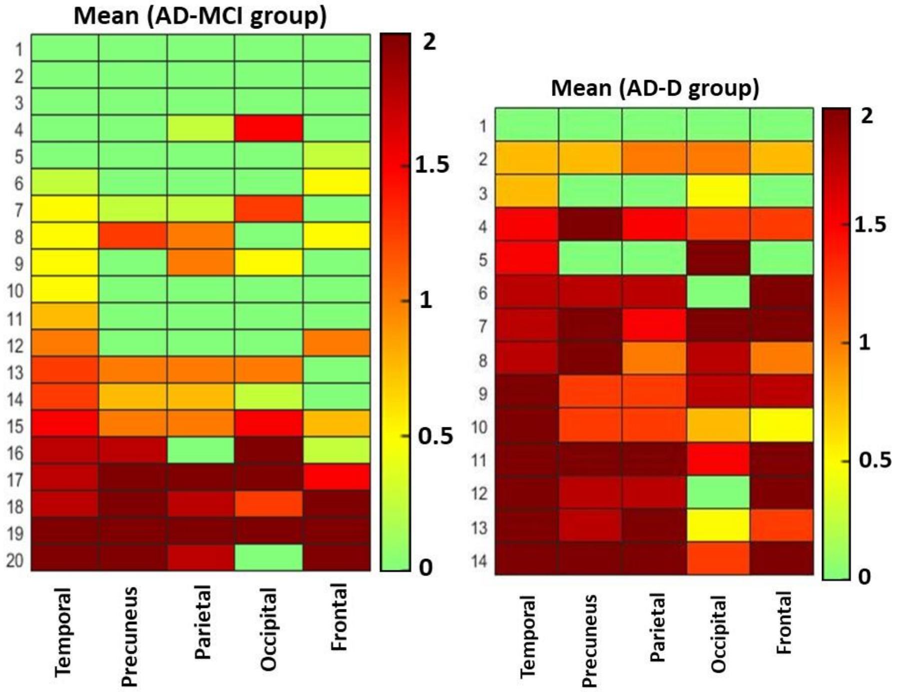

sFigure 4. This illustrates the heatmaps of the mean regional reading scores for both AD-MCI (left) and AD-D groups. Most patients exhibit typical temporoparietal uptake pattern but some atypical pattern can be observed. For example, case 12 in the AD-MCI group and cases 3 and 5 in the AD-D group are with different uptake patterns from the typical temporoparietal one.

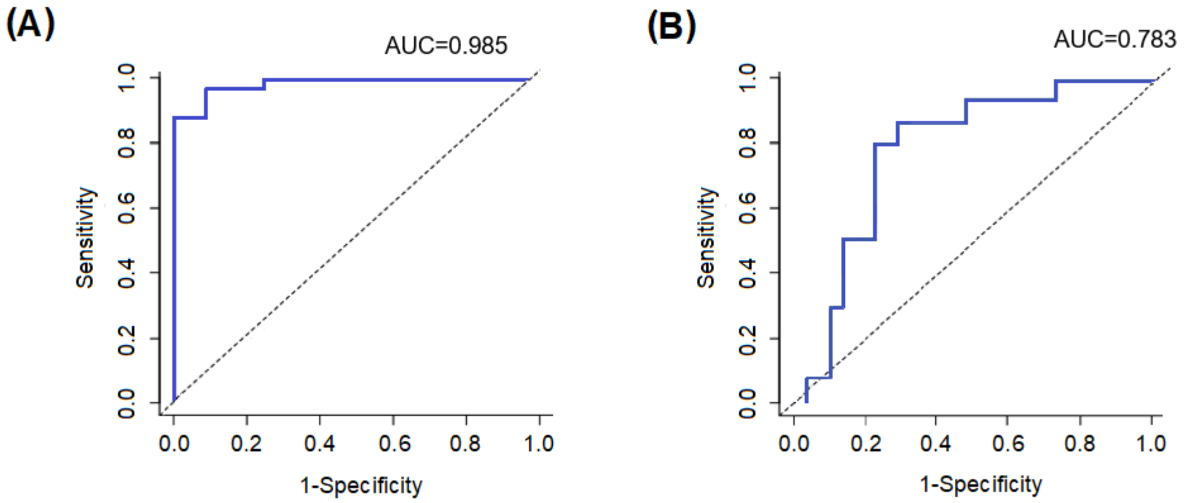

sFigure 5. This shows ROC analyses for the global SUVr to (A) distinguish CU (n=12) versus all other participants (n=34) (AUC =0.985), and (B) distinguish AD-D (n=14) versus all other participants (n=32) (AUC=0.783). CU: cognitively unimpaired subjects; AD-D: Alzheimer's disease with dementia. AUC: area under the ROC.
